# Tratamento Medicamentoso da Hipertensão: Do Trio de Ouro ao Octeto

**DOI:** 10.36660/abc.20190780

**Published:** 2020-08-19

**Authors:** Audes Diógenes Magalhães Feitosa, Marco Mota-Gomes, Oswaldo Passarelli, Weimar Kunz Sebba Barroso, Roberto Dischinger Miranda, Eduardo Costa Duarte Barbosa, Andrea A. Brandão, Wilson Nadruz

**Affiliations:** 1 Unidade de Hipertensão e Cardiologia Preventiva PROCAPE Universidade de Pernambuco Recife PE Brasil Unidade de Hipertensão e Cardiologia Preventiva, PROCAPE, Universidade de Pernambuco, Recife, PE – Brasil; 2 Centro Universitário CESMAC Maceió AL Brasil Centro Universitário CESMAC, Maceió, AL - Brasil; 3 Instituto Dante Pazzanese de Cardiologia São Paulo SP Brasil Instituto Dante Pazzanese de Cardiologia,São Paulo, SP – Brasil; 4 Universidade Federal de Goiás Liga de Hipertensão Goiânia GO Brasil Universidade Federal de Goiás - Liga de Hipertensão, Goiânia, GO - Brasil; 5 Universidade Federal de São Paulo Escola Paulista de Medicina São Paulo SP Brasil Universidade Federal de São Paulo - Escola Paulista de Medicina, São Paulo, SP - Brasil; 6 Liga de Hipertensão de Porto Alegre Porto Alegre RS Brasil Liga de Hipertensão de Porto Alegre, Porto Alegre, RS – Brasil; 7 Universidade do Estado do Rio de Janeiro Rio de Janeiro RJ Brasil Universidade do Estado do Rio de Janeiro, Rio de Janeiro, RJ - Brasil; 8 Universidade Estadual de Campinas Campinas SP Brasil Universidade Estadual de Campinas, Campinas, SP – Brasil

**Keywords:** Hipertensão, Anti-Hipertensivos, Tratamento Farmacológico, Estilo de Vida, Exercício, Perda de Peso, Adesão à Medicação

O tratamento da hipertensão arterial (HA) envolve diversas opções medicamentosas, o que pode dificultar a uniformização de condutas, contribuindo assim para o insucesso terapêutico.^1^ Nos últimos anos, entretanto, diversos estudos e diretrizes de HA de várias sociedades sugeriram classes medicamentosas preferenciais para o tratamento da HA.^2 - 6^ Com base nessas evidências, o presente artigo tem como objetivo propor um algoritmo de tratamento medicamentoso simples e prático que possa ser utilizado em pacientes que tenham desde HA estágio 1 até HA refratária ( Figura 1 ).

**Figura 1:**
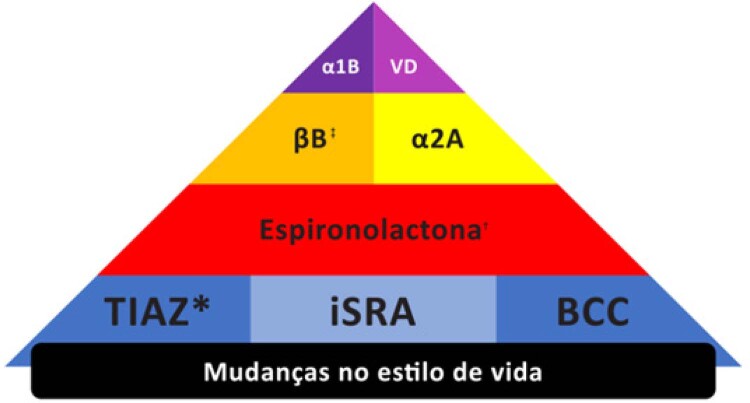
– *O octeto medicamentoso para tratamento da hipertensão arterial.* TIAZ: diurético tiazídico/tipo tiazídico; iSRA: inibidor do sistema renina-angiotensina; BCC: bloqueador do canal de cálcio; βB: betabloqueador; α2A: agonista alfa-2 central; α1B: bloqueador alfa-1 adrenérgico; VD: vasodilatador arterial direto. *Caso não haja controle da pressão arterial com TIAZ, iSRA e BCC, e o TIAZ for hidroclorotiazida, substituir o TIAZ por clortalidona ou indapamida. Se a taxa de filtração glomerular for <30 mL/min, substituir TIAZ por diurético de alça, como furosemida. †Se não houver tolerância à espironolactona, especialmente por efeitos antiandrogênicos, considerar substituir esta medicação por amilorida. ‡βB está indicado como escolha inicial caso existam indicações específicas, tais como angina, pós-infarto do miocárdio, insuficiência cardíaca, arritmia ou controle da frequência cardíaca.

O tratamento combina mudanças no estilo de vida (incluindo a redução da ingestão de sódio, controle do peso, realização de atividade física, moderação no consumo de álcool e abolição do tabagismo), descontinuação de substâncias que desencadeiam hipertensão e adição sequencial de anti-hipertensivos.^2 - 4 , 7 , 8^ De acordo com as diretrizes atuais de HA, as classes anti-hipertensivas a serem preferencialmente iniciadas no tratamento do paciente hipertenso incluem o chamado *trio de ouro:* ^9^ um inibidor do sistema renina-angiotensina (iSRA) (inibidor da enzima conversora de angiotensina ou um bloqueador do receptor de angiotensina II), um bloqueador do canal de cálcio (BCC) ou um diurético tiazídico/tipo tiazídico (TIAZ).^2 - 4^ O início do tratamento na maioria dos pacientes compreende dois medicamentos, com o intuito de otimizar a eficiência e a previsibilidade do controle da pressão arterial (PA). Por outro lado, a monoterapia está reservada para pacientes de baixo risco com HA em estágio 1, pacientes pré-hipertensos de alto risco ou pacientes idosos frágeis.^2 , 4^ As combinações de dois medicamentos habitualmente preferidas são um iSRA com um BCC ou um iSRA com um TIAZ,^4^ embora em pacientes com alto risco cardiovascular a combinação de iSRA com BCC pareça ser superior à combinação de iSRA com TIAZ na redução de desfechos cardiovasculares.^10^ Caso a meta pressórica preconizada não seja atingida com dois medicamentos, o uso de três fármacos deve compreender preferencialmente os componentes do *trio de ouro.* Se a PA não for controlada com o uso dessas 3 classes, sendo a hidroclorotiazida o TIAZ utilizado, o controle pode ser melhorado substituindo-se a hidroclorotiazida por outro TIAZ de longa ação mais potente (clortalidona ou indapamida).^1 , 11^ Além disso, um diurético de alça, como a furosemida, deve substituir o TIAZ se a taxa de filtração glomerular for <30 mL/min.^11^

Os betabloqueadores (βB), que no passado eram considerados classes iniciais preferenciais,^12 , 13^ perderam espaço como primeira escolha no tratamento da HA de acordo com as diretrizes mais recentes. Assim, os βB estão indicados como monoterapia ou em combinação com outras medicações quando existirem indicações específicas, tais como angina, pós-infarto do miocárdio, insuficiência cardíaca, arritmia ou controle da frequência cardíaca.^2 - 4^

O controle inadequado da PA mesmo com o uso de 3 classes de fármacos deve ser confirmado pela medida ambulatorial da PA (MAPA) ou medida residencial da PA (MRPA) e após exclusão de causas de HA pseudorresistente (principalmente baixa adesão à medicação e posologia inadequada).^1 , 11 , 14^ Pacientes que estão com PA não controlada usando doses máximas de 3 ou mais classes de medicações, incluindo iSRA, BCC e TIAZ, em quem a pseudorresistência foi excluída, são considerados portadores de HA resistente. Além disso, aqueles tomando 4 classes de medicações, incluindo iSRA, BCC e TIAZ, e que estejam com PA controlada, são considerados como hipertensos resistentes controlados. Já aqueles com PA não controlada usando doses máximas de 5 ou mais classes de medicações, incluindo TIAZ de longa ação e espironolactona, são considerados portadores de HA refratária. Nos casos de HA resistente ou refratária, é necessária a realização de exames complementares para investigação de lesão em órgãos-alvo e causas secundárias de HA e, se necessário, estabelecer um tratamento específico para a causa secundária.

Evidências crescentes sugerem que, na ausência de controle de PA com uso otimizado e concomitante de iSRA, BCC e TIAZ, a quarta classe anti-hipertensiva a ser utilizada deve envolver o bloqueio da aldosterona por meio do uso de baixas doses de um antagonista de receptor mineralocorticoide, como a espironolactona 25 ou 50 mg/dia, conforme mostrado em diversos estudos e em metanálises.^5 , 15 - 17^ Contudo, nem todos os pacientes toleram a espironolactona devido especialmente a efeitos colaterais antiandrogênicos, resultando em sensibilidade mamária ou ginecomastia, disfunção erétil nos homens e irregularidades menstruais nas mulheres. Nesse contexto, resultados do estudo PATHWAY-2 sugerem que a amilorida, um diurético poupador de potássio, na dose de 10 ou 20 mg/dia, é tão eficaz quanto a espironolactona na redução da PA, podendo constituir uma opção terapêutica em substituição à espironolactona nos pacientes com HA resistente.^15^ Entretanto, é válido ressaltar que a amilorida, de forma isolada e na dosagem citada, não é disponível atualmente no Brasil.

O estudo ReHOT comparou os efeitos da espironolactona com um agonista alfa-2 central (clonidina) em hipertensos resistentes. Embora não tenham sido encontradas diferenças no desfecho primário (taxa de controle da PA no consultório ou da MAPA) entre a espironolactona e a clonidina, resultados de análises secundárias mostraram reduções maiores na PA de 24 horas com a espironolactona, reforçando a opção da espironolactona como o quarto fármaco preferencial no tratamento da HA resistente.^6^ Contudo, as reduções de PA com clonidina também foram substanciais, e colocam esta medicação também como uma boa opção a ser acrescentada à espironolactona caso não haja controle da PA.

O estudo PATHWAY-2 também investigou o uso de um βB (bisoprolol) ou um bloqueador alfa-1 adrenérgico (doxazosina) como alternativas à espironolactona. Essas medicações não foram tão eficazes quanto a espironolactona, mas reduziram significativamente a PA *versus* o placebo quando adicionadas ao tratamento de base na HA resistente.^5^ Deste modo, devem ser acrescentadas posteriormente à espironolactona. Como o estudo ALLHAT havia mostrado que a monoterapia com doxazosina foi substancialmente inferior à monoterapia com clortalidona na prevenção de eventos cardiovasculares, especialmente insuficiência cardíaca,^18^ consideramos que um bloqueador alfa-1 adrenérgico deva ser uma das últimas escolhas para o tratamento do hipertenso resistente.

Por fim, poucos estudos avaliaram o impacto anti-hipertensivo de vasodilatadores diretos, como hidralazina ou minoxidil, na HA resistente. No entanto, como esta classe pode provocar retenção de líquidos e taquicardia de forma muitas vezes exuberante, também fica habitualmente reservada como uma das últimas escolhas a ser considerada no tratamento da HA resistente.^11^

Em resumo, com base nas evidências supracitadas, propomos um octeto medicamentoso estruturado para tratamento da HA ( Figura 1 ). Na base do tratamento de todo o paciente hipertenso estão as mudanças no estilo de vida e os componentes do *trio de ouro* (iSRA, BCC e TIAZ). A espironolactona deve ser preferencialmente utilizada como quarta substância, caso não haja controle com as medicações anteriores. Posteriormente, podem ser acrescentados agonistas alfa-2 centrais e βB, sendo que as últimas medicações a serem acrescentadas seriam os vasodilatadores e os bloqueadores alfa-1 adrenérgicos.

## References

[1] 1. Acelajado MC, Hughes ZH, Oparil S, Calhoun DA. Treatment of resistant and refractory hypertension. Circ Res. 2019;124(7):1061-70. 10.1161/CIRCRESAHA.118.312156 PMC6469348 30920924

[2] 2. Malachias MVB, Jardim PCV, Almeida FA, Lima Jr E, Feitosa GS. 7th Brazilian Guideline of Arterial Hypertension: Chapter 7 - Pharmacological Treatment. Arq Bras Cardiol. 2016;107(3 Suppl 3):35-43. 10.5935/abc.20160157 PMC5319469 27819386

[3] 3. Whelton PK, Carey RM, Aronow WS, Casey DE Jr, Collins KJ, Dennison Himmelfarb C, et al. 2017 ACC/AHA/AAPA/ABC/ACPM/AGS/APhA/ASH/ASPC/NMA/PCNA Guideline for the Prevention, Detection, Evaluation, and Management of High Blood Pressure in Adults: Executive Summary: A Report of the American College of Cardiology/American Heart Association Task Force on Clinical Practice Guidelines. J Am Coll Cardiol. 2018;71:2199-2269.

[4] 4. Williams B, Mancia G, Spiering W, Agabiti Rosei E, Azizi M, Burnier M, et al. 2018 ESC/ESH Guidelines for the management of arterial hypertension. Eur Heart J. 2018;39(33):3021-3104. 10.1093/eurheartj/ehy339 30165516

[5] 5. Williams B, MacDonald TM, Morant S, Webb DJ, Sever P, McInnes G, et al. Spironolactone versus placebo, bisoprolol, and doxazosin to determine the optimal treatment for drug-resistant hypertension (PATHWAY-2): a randomised, double-blind, crossover trial. Lancet. 2015;386(1008):2059–68. 10.1016/S0140-6736(15)00257-3 PMC4655321 26414968

[6] 6. Krieger EM, Drager LF, Giorgi DMA, Pereira AC, Barreto-Filho JAS, Nogueira AR, et al. Spironolactone versus clonidine as a fourth-drug therapy for resistant hypertension: the ReHOT randomized study (Resistant Hypertension Optimal Treatment). Hypertension. 2018;71:681–90. 10.1161/HYPERTENSIONAHA.117.10662 29463627

[7] 7. Pimenta E, Gaddam KK, Oparil S, Aban I, Husain S, Dell’Italia LJ, et al. Effects of dietary sodium reduction on blood pressure in subjects with resistant hypertension: results from a randomized trial. Hypertension. 2009;54(3):475–81. 10.1161/HYPERTENSIONAHA.109.131235 PMC2771382 19620517

[8] 8. Précoma DB, Oliveira GMM, Simão AF, Dutra OP, Coelho OR, Izar MCO, et al. Atualização da Diretriz de Prevenção Cardiovascular da Sociedade Brasileira de Cardiologia – 2019. Arq Bras Cardiol. 2019;113(4):787-891.

[9] 9. Passarelli Jr O. Resistant hypertension: how I treat. Rev Bras Hipertens. 2011;18(4):160-2.

[10] 10. Jamerson K, Weber MA, Bakris GL, Dahlöf B, Pitt B, Shi V, et al. Benazepril plus amlodipine or hydrochlorothiazide for hypertension in high-risk patients. N Engl J Med. 2008 Dec 4;359:2417-28. 10.1056/NEJMoa0806182 19052124

[11] 11. Carey RM, Calhoun DA, Bakris GL, Brook RD, Daugherty SL, Dennison-Himmelfarb CR, et al. Resistant hypertension: detection, evaluation, and management: a scientific statement from the American Heart Association. Hypertension. 2018;72:e53-e90. 10.1161/HYP.0000000000000084 PMC6530990 30354828

[12] 12. Mancia G, Fagard R, Narkiewicz K, Redon J, Zanchetti A, Böhm M, et al. 2013 ESH/ESC Guidelines for the management of arterial hypertension: the task force for the management of arterial hypertension of the European Society of Hypertension (ESH) and of the European Society of Cardiology (ESC). Eur Heart J. 2013;34(28):2159-2219. 10.1093/eurheartj/eht151 23771844

[13] 13. Tavares A, Brandão AA, Sanjuliani AF, Nogueira AR, Machado CA, Poli-de-Figueiredo E, et al. VI Diretrizes Brasileiras de Hipertensão Arterial. Arq Bras Cardiol. 2010;95(1 supl 1):1-51. 21085756

[14] 14. Alessi A, Brandão AA, Coca A, Cordeiro AC, Nogueira AR, Diógenes de Magalhães F, et al. First Brazilian position on resistant hypertension. Arq Bras Cardiol. 2012;99(1):576-85. 10.1590/s0066-782x2012000700002 22948302

[15] 15. Williams B, MacDonald TM, Morant SV, Webb DJ, Sever P, McInnes GT, et al. Endocrine and haemodynamic changes in resistant hypertension, and blood pressure responses to spironolactone or amiloride: the PATHWAY-2 mechanisms substudies. Lancet Diabetes Endocrinol. 2018;6(6):46475. 10.1016/S2213-8587(18)30071-8 PMC5966620 29655877

[16] 16. Liu L, Xu B, Ju Y. Addition of spironolactone in patients with resistant hypertension: a meta-analysis of randomized controlled trials. Clin Exp Hypertens. 2017;39(3):257-63. 10.1080/10641963.2016.1246564 28448185

[17] 17. Zhao D, Liu H, Dong P, Zhao J. A meta-analysis of add-on use of spironolactone in patients with resistant hypertension. Int J Cardiol. 2017 Apr 15;233:113-17. 10.1016/j.ijcard.2016.12.158 28089457

[18] 18. The ALLHAT Officers and Coordinators for the ALLHAT Collaborative Research Group. Major cardiovascular events in hypertensive patients randomized to doxazosin vs chlorthalidone: the Antihypertensive and Lipid-Lowering Treatment to Prevent Heart Attack Trial (ALLHAT). JAMA. 2000;283(15):1967–75. 10789664

