# Supplementary material for: Tratamento Medicamentoso da Hipertensão: Do Trio de Ouro ao Octeto
Source: Arq Bras Cardiol. 2020 Aug 19;115(2):270–2. [Article in Portuguese] doi: 10.36660/abc.20190780 (PMC8384273; doi:10.36660/abc.20190780)
Supplement: Translation

# Translation: Pharmacological Treatment of Hypertension: From the Golden Trio to the Octet

**Authors:** Audes Diógenes Magalhães Feitosa, Marco Mota-Gomes, Oswaldo Passarelli, Weimar Kunz Sebba Barroso, Roberto Dischinger Miranda, Eduardo Costa Duarte Barbosa, Andrea A. Brandão, Wilson Nadruz

**Affiliations:** 1 Brazil Unidade de Hipertensão e Cardiologia Preventiva, PROCAPE, Universidade de Pernambuco, Recife, PE – Brazil; 2 Brazil Centro Universitário CESMAC, Maceió, AL - Brazil; 3 Brazil Instituto Dante Pazzanese de Cardiologia, São Paulo, SP – Brazil; 4 Brazil Universidade Federal de Goiás - Liga de Hipertensão, Goiânia, GO - Brazil; 5 Brazil Universidade Federal de São Paulo - Escola Paulista de Medicina, São Paulo, SP - Brazil; 6 Brazil Liga de Hipertensão de Porto Alegre, Porto Alegre, RS – Brazil; 7 Brazil Universidade do Estado do Rio de Janeiro, Rio de Janeiro, RJ - Brazil; 8 Brazil Universidade Estadual de Campinas, Campinas, SP – Brazil

**Keywords:** Hypertension, Antihypertensive Agents, Drug Therapy, Life Style, Exercise, Weight Loss, Medication Adherence

The treatment of hypertension comprises numerous pharmacological options, which may hinder patient management standardization, thus contributing to therapeutic failure.^1^ However, in more recent years, several studies and guidelines from diverse hypertension and cardiology societies have suggested preferential pharmacological classes to treat hypertension.^2 - 6^ Based on this evidence, the present report aims to propose a simple and practical pharmacological treatment algorithm that can be applied to patients ranging from stage 1 to refractory hypertension cases ( Figure 1 ).

**Figure 1:** – *Structured octet for the treatment of hypertension.* THIAZ: thiazide-type/thiazide-like diuretic; RASI: renin angiotensin system inhibitor; CCB: calcium-channel blocker; βB: Beta-blocker; α2A: central alpha-2 agonist; α1B: alpha-1 adrenergic blocker; VD: direct vasodilator. *When BP control is not achieved with THIAZ, RASI and CCB, and the THIAZ is hydrochlorothiazide, substitute this latter drug by a long-acting THIAZ (chlortalidone or indapamide). If glomerular filtration rate <30 mL/min, substitute THIAZ by a loop diuretic, such as furosemide. †If spironolactone is not tolerated, particularly due to anti-androgenic side effects, consider substituting this drug by amiloride. ‡βB is indicated as the first choice for the initial treatment when there are specific indications, such as angina, post-myocardial infarction, heart failure, arrhythmia or heart rate control.

Hypertension treatment combines lifestyle changes (including salt intake reduction, weight control, physical activity performance , alcohol intake moderation, and smoking cessation), discontinuation of substances that may increase blood pressure (BP), and sequential addition of antihypertensive medications.^2 - 4 , 7 , 8^ According to current hypertension guidelines, antihypertensive classes that should be preferentially initiated for the treatment of hypertensive patients include the so-called *golden trio* :^9^ a renin-angiotensin system inhibitor (RASI) (angiotensin-converting enzyme inhibitor or angiotensin II receptor blocker), a calcium-channel blocker (CCB) or a thiazide-type/thiazide-like diuretic (THIAZ).^2 - 4^ In most hypertensive patients, treatment initiation usually includes the combination of two pharmacological classes, an approach that aims to optimize BP control efficiency and predictability. Conversely, monotherapy has been recommended for stage 1 hypertensive patients with low cardiovascular risk, pre-hypertensive patients and frail elderly patients.^2 , 4^ The usual preferred dual combinations comprise a RASI plus a CCB or a RASI plus a THIAZ,^4^ although in patients with high cardiovascular risk the combination of RASI plus CCB seems to be superior to the combination of RASI plus THIAZ in reducing adverse cardiovascular events.^10^ If BP control is not achieved with two pharmacological classes, the use of three drugs should be instituted, preferentially comprising the *golden trio* components. When these three drugs are used, but BP control is not achieved and hydrochlorothiazide is the prescribed THIAZ, this latter drug should be substituted by a long-acting THIAZ (chlortalidone or indapamide).^1 , 11^ In addition, a loop diuretic, such as furosemide, should replace the THIAZ if glomerular filtration rate is <30 mL/min.^11^

Beta-blockers (βB), which were considered a preferential initial class for hypertension treatment in the past,^12 , 13^ have not been recommended as a first-choice class to treat hypertension according to more recent guidelines. Therefore, βB have been indicated as monotherapy or in combination with other classes when there are specific indications, such as angina, post-myocardial infarction, heart failure, arrhythmia or heart rate control.^2 - 4^

The inadequate control of BP with the use of three drug classes should be confirmed by ambulatory or home BP monitoring, after excluding causes of pseudo-resistant hypertension (mainly poor medication adherence and inadequate dosage).^1 , 11 , 14^ Patients with uncontrolled BP using maximal dosages of three or more antihypertensive classes, including RASI, CCB and THIAZ, and in which pseudo-resistance was ruled out, are considered as having resistant hypertension, whereas those who have controlled BP while taking four antihypertensive classes, including RASI, CCB and THIAZ, are considered as having controlled resistant hypertension. Patients with uncontrolled BP using maximal dosages of five or more antihypertensive classes, including a long-acting THIAZ and spironolactone, are considered as having refractory hypertension. It is noteworthy that patients with resistant or refractory hypertension should undergo further investigation of end-organ damage and investigation/treatment of secondary causes of hypertension.

Growing evidence has suggested that, in the absence of BP control with optimized and concomitant use of RASI, CCB and THIAZ, the fourth antihypertensive class to be instituted should be an aldosterone antagonist, particularly low-dose spironolactone (25-50 mg/day), as demonstrated in several studies and meta-analyses.^5 , 15 - 17^ However, spironolactone may not be tolerated by some patients, due to its anti-androgenic side effects, resulting in gynecomastia or breast tenderness, impotence in men, and menstrual irregularities in women. In this regard, the results of the PATHWAY-2 trial suggested that 10-20 mg/day of amiloride, a potassium-sparing diuretic, is as effective as spironolactone in reducing BP in resistant hypertensive patients, thus constituting an alternative to spironolactone in the treatment of resistant hypertension.^15^ However, it should be noted that amiloride, as an isolated formulation and at the aforementioned dosage, is not currently available in Brazil.

The ReHOT study compared the effects of spironolactone *versus* a central alpha-2 agonist (clonidine) in resistant hypertensive patients. Although there were no differences in the primary endpoint (BP control during office and ambulatory BP monitoring) achieved by spironolactone or clonidine, results of the secondary analysis showed greater 24-hour BP reduction with spironolactone, reinforcing the use of spironolactone as the fourth preferential drug for the treatment of resistant hypertension.^6^ However, BP reductions achieved by clonidine were also substantial, which may establish this drug as a good option to be added to spironolactone when BP control has not been attained.

The PATHWAY-2 trial also investigated the BP effects of a βB (bisoprolol) or an alpha-1 adrenergic blocker (doxazosin) as alternative medications to spironolactone. These drugs were not as effective as spironolactone, but significantly reduced BP *versus* placebo when added to baseline antihypertensive medications in resistant hypertensive patients.^5^ Therefore, a βB or an alpha-1 adrenergic blocker should be added subsequently to spironolactone in patients with uncontrolled BP. However, because the ALLHAT study showed that doxazosin was markedly inferior to chlortalidone in preventing cardiovascular events, particularly heart failure,^18^ we suggest that an alpha-1 adrenergic blocker should be one of the last antihypertensive classes to be added to the treatment of patients with resistant hypertension.

Few studies have evaluated the impact of direct vasodilators, such as hydralazine or minoxidil, in the treatment of resistant hypertension. In addition, this antihypertensive class may cause marked fluid retention and tachycardia, and therefore should be considered as one of last choices in the treatment of resistant hypertension.^11^

In summary, based on the abovementioned data, we propose a structured octet for the pharmacological treatment of hypertension ( Figure 1 ). Lifestyle changes and components of the *golden trio* (RASI, CCB and THIAZ) comprise the bottom of the treatment algorithm. Spironolactone should be preferentially used as the fourth antihypertensive class when there is no adequate BP control with the latter medications. Then, central alpha-2 agonists and βB may be added, while direct vasodilators and alpha-1 adrenergic blockers should be considered as the last options to be instituted for hypertension treatment.
